# Discrimination between Alzheimer's Disease and Mild Cognitive Impairment Using SOM and PSO-SVM

**DOI:** 10.1155/2013/253670

**Published:** 2013-05-07

**Authors:** Shih-Ting Yang, Jiann-Der Lee, Tzyh-Chyang Chang, Chung-Hsien Huang, Jiun-Jie Wang, Wen-Chuin Hsu, Hsiao-Lung Chan, Yau-Yau Wai, Kuan-Yi Li

**Affiliations:** ^1^Department of Electrical Engineering, Chang Gung University, Tao-Yuan 333, Taiwan; ^2^Department of Occupational Therapy, Bali Psychiatric Center, New Taipei City 249, Taiwan; ^3^Department of Medical Imaging and Radiological Sciences, Chang Gung University, Tao-Yuan 333, Taiwan; ^4^Department of Neuroscience, Chang Gung Memorial Hospital, Tao-Yuan 333, Taiwan; ^5^Chang Gung Dementia Center, Chang Gung Memorial Hospital, Tao-Yuan 333, Taiwan; ^6^Department of Medical Imaging and Intervention, Chang Gung Memorial Hospital, Tao-Yuan 333, Taiwan; ^7^Department of Occupational Therapy, Chang Gung University, Tao-Yuan 333, Taiwan

## Abstract

In this study, an MRI-based classification framework was proposed to distinguish the patients with AD and MCI from normal participants by using multiple features and different classifiers. First, we extracted features (volume and shape) from MRI data by using a series of image processing steps. Subsequently, we applied principal component analysis (PCA) to convert a set of features of possibly correlated variables into a smaller set of values of linearly uncorrelated variables, decreasing the dimensions of feature space. Finally, we developed a novel data mining framework in combination with support vector machine (SVM) and particle swarm optimization (PSO) for the AD/MCI classification. In order to compare the hybrid method with traditional classifier, two kinds of classifiers, that is, SVM and a self-organizing map (SOM), were trained for patient classification. With the proposed framework, the classification accuracy is improved up to 82.35% and 77.78% in patients with AD and MCI. The result achieved up to 94.12% and 88.89% in AD and MCI by combining the volumetric features and shape features and using PCA. The present results suggest that novel multivariate methods of pattern matching reach a clinically relevant accuracy for the a priori prediction of the progression from MCI to AD.

## 1. Introduction

Alzheimer's disease (AD) [1] is the most common type of dementia. Clinical signs are characterized by progressive cognitive deterioration, together with declining activities of daily living and by neuropsychiatric symptoms or behavioral changes. The early detection of AD is potentially challenging because of several reasons. First of all, there existed no known biomarkers. The disease usually has an insidious onset which can be a combination of genetic and environmental factors. It is difficult to differentiate other types of dementia.

Mild cognitive impairment (MCI) is a transitional stage between normal aging and demented status. The syndrome is defined by the greater cognitive decline than age and education matched individuals, but no interference of daily function [2]. According to the major symptoms, MCI is characterized with memory loss and cognitive impairment. Research has reported that MCI has a risk between 10% to 64% developing AD [3, 4]. AD is a progressively neuro-degenerative disorder and is distinguished from MCI by the progressive deterioration of daily function. The prevalence of AD increases dramatically at age 65 and it affects approximately 26 million people worldwide, which may increase fourfolds by the year of 2050. Recent reports in the treatment or prevention of AD lead to a growing concerns in the early diagnosis. Therefore, the detection of changes in brain tissues that reflect the pathological processes of MCI would prevent or postpone the disease progresses either from normal control to MCI or from MCI to AD. If MCI can be diagnosed at an early stage and effectively intervened, then it is possible to reduce the advanced damages.

Since the poor performance in memory and execution function indicates the high risk of dementia, the probable AD patients are usually evaluated by standardized neuropsychological tests [5–8]. Additionally, many studies have been proposed to examine the predictive abilities of nuclear imaging with respect to AD and other dementia illnesses [9–13]. However, under the consideration of imaging cost and noninvasive requirement, magnetic resonance imaging (MRI) has been widely used for early detection and diagnosis of MCI and AD [14–17].

Atrophy typically starts in the medial temporal and limbic areas, subsequently extending to parietal association areas, and finally to frontal and primary cortices. Early changes in hippocampus and entorhinal cortex have been demonstrated with the help of MRI, and these changes are consistent with the underlying pathology of MCI and AD. Many studies have used manual or automatic methods to measure hippocampus and entorhinal cortex [18–20]. Hippocampal volumes and entorhinal cortex measures have been found to be equally accurate in distinguishing between AD and normal cognitive elderly subjects [21]. However, the segmentation and identification of hippocampus or entorhinal cortex are usually sensitive to the subjective opinion of the operator and also time consuming. In addition, the enlargement of ventricles is also a significant characteristic of AD due to neuronal loss. Ventricles are filled with cerebrospinal fluid (CSF) and surrounded by gray matter (GM) and white matter (WM). As a result, by measuring the ventricular enlargement, hemispheric atrophy rate shows higher correlation with the disease progression.

In this study, we have designed an MRI-based classification framework to distinguish the patients of MCI and AD from normal individuals using multiple features and different classifiers. Since the features adopted here are volume-related and shape-related, we also aimed to investigate whether the combination of both statistical analysis and principal component analysis (PCA) would improve the accuracies of classification than using volume-related alone, shape-related alone, or all features. Our hypothesis was that the combination of all MRI-based features is helpful for distinguishing the patients with early Alzheimer's disease from the subjects with mild cognitive impairment and healthy controls, respectively.

The remainder of this paper is organized as follows.  Section 2 illustrated the proposed scheme, including features extraction and used classifiers, that is, self-organizing map (SOM), support vector machine (SVM), particle swarm optimization (PSO), and the proposed hybrid PSO-SVM. Statistical analysis, experimental results, and discussion are revealed in Section 3. Finally, conclusions are included in Section 4.

## 2. The Proposed Schemes

**Figure 1:**
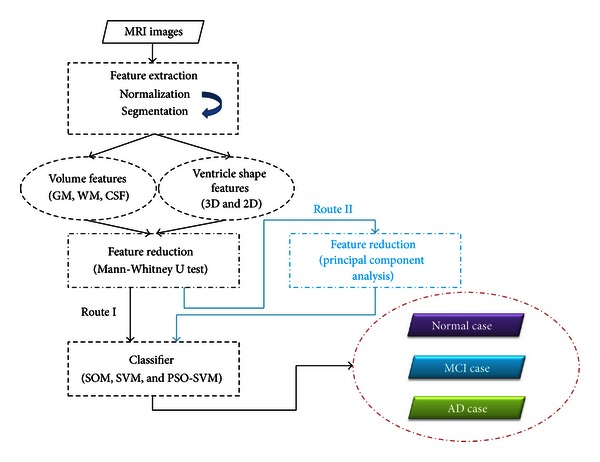
Flowchart of the proposed image-aided diagnosis system.

Figure 1 is the flowchart that demonstrated the system we proposed. In the step of *Feature Extraction*, spatial normalization is performed by coregistering the brain MRI data from each individual to a T1-weighted MRI template such that these images of the investigated subjects will be in the same scale space. Next, with the aids of segmentation and morphological procedures, all MRI brain images are segmented into GM, WM, CSF, and ventricle's tissues and shape descriptors. Here, volume-related and shape-related features are utilized for further classification. The step of *Feature Reduction* is divided into two parts: (1) Mann-Whitney *U* test is adopted to filter out the features with low discriminative power; (2) principal component analysis (PCA) is applied to reduce the dimensions of feature space. Route I only uses *U* test; Route II is combined with *U* test and PCA. At last, a classifier, for example, SOM, SVM, and PSO-SVM, is employed to classify tested volunteers into three categories: normal individuals, MCI, and AD patients. The details of the proposed method are described below.

### 2.1. Spatial Normalization of MRI Data

Spatial normalization of the brain images is useful for determining what happens generically over individuals. It is a procedure to register an MRI data set to a standard coordinate system, also known as Talairach and Tournoux coordinate system [22]. With the aid of normalization, all images were spatially normalized to stereotactic space ICBM-152 [23] via a 12-degrees-of-freedom affine transformation which normalizes the brain in terms of dimensions, position, and spatial orientation.

### 2.2. Volume Features Extraction

**Figure 2:**
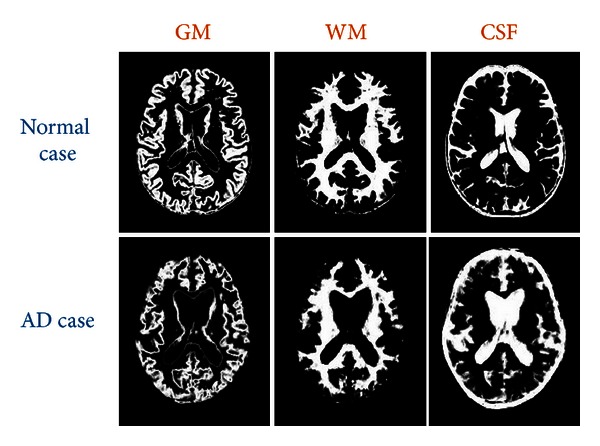
Segmentation results of a normal individual and an AD patient used in this study.

The volumes of brain tissues such as GM, WM, and CSF indicate important information, especially in brain degeneration diseases [24]. A clustering-based segmentation algorithm provided by SPM8 [25] is using a modified Gaussian mixture model to extract GM, WM and CSF probability maps from whole-brain MRI data. The intensities of voxels belonging to each of these clusters conform to a normal distribution which can be described by a mean, a variance, and the number of voxels belonging to the distribution. Here, the volumes of GM, WM, CSF, and whole-brain are calculated by

(1) $$\begin{array}{c} \text{volum}{\text{e}}_{\text{tissue}}\approx \sum_{\forall i\in I}\left(P\left(C_{\text{tissue}}\;|\;f\left(i\right)\right)>0.5\right),\\ \text{volum}{\text{e}}_{\text{Whole}}\approx \sum_{\forall i\in I}\left(P\left(C_{\text{GM}\vee \text{WM}}\;|\;f\left(i\right)\right)>0.5\right), \end{array}$$

where *i* is any pixel of the MRI data and *f*(*i*) stands for the gray level of *i*. *C* means the cluster. tissue stands for the parts of GM, WM, or CSF.  Figure 2 illustrates the segmentation results of the normal individual and AD patient used in this study.

Next, we employ region growing and double threshold algorithm [26] to extract binary ventricle volume data, that is, *M*(*x*, *y*, *z*). The morphological operators, for example, erosion and dilation, are used to obtain the binary ventricle regions. And the edges of binary images are detected by applying Sobel operation on a slice-by-slice basis. Then, this segmented region will construct a binary mask image. In this mask image, 1 (white) denotes the ventricle pixel, and 0 (black) denotes the nonventricle pixel. Finally, we can calculate the volume of cerebral ventricle by

(2) $$\begin{array}{c} \text{volum}{\text{e}}_{\text{Ventricle}}\approx \sum_{\forall i\in M}\left(P\left(C_{\text{Ventricle}}\;|\;f\left(i\right)\right)=1\right), \end{array}$$

where *i* is any pixel of the mask data, *M* is the mask image, and *f*(*i*) denotes the gray level of *i*.

### 2.3. Shape Features Extraction

**Figure 3:**
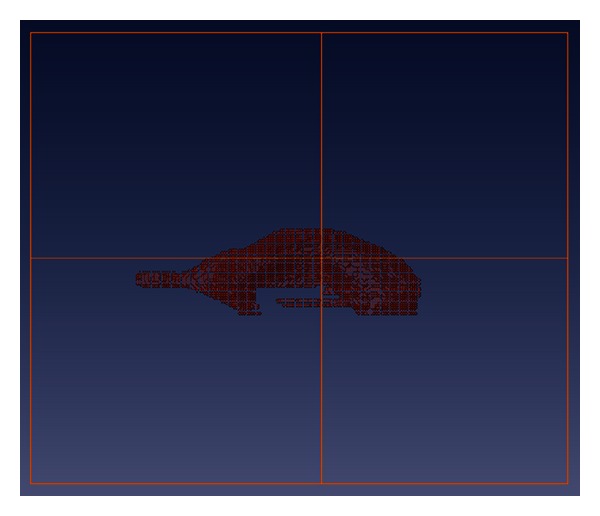
Sagittal view of segmented ventricle.

The volume features, which are extracted from the whole three dimensional volume, cannot capture the variation of the anatomical shape. Wang et al. [27, 28] proposed a ventricle shape-based method for improved classification of Alzheimer's patients. Therefore, to enhance the accuracy of the classification, in addition to the volume features, we also added ventricle shape features.  Figure 3 shows the sagittal view of ventricle that we segmented. The shape features we analyzed are composed of two types: three-dimensional shape features and two-dimensional shape features. The algorithms to obtain these features are illustrated in the following subsections.

#### 2.3.1. D Shape Features

To obtain the feature of 3D shape, a leave-one-out method is used to construct training set and testing set following Wang's method. Three sets of probability map were then built using

(3) $$\begin{array}{c} P_{t}\left(x,y,z\right)=\frac{1}{N}\sum_{i=1}^{N}I_{t}^{i}\left(x,y,z\right), \end{array}$$

where *t* indicates the type of the subjects, inclusive of normal control, AD, and MCI. *N* is the number of training samples, and *I* denotes the gray level of the ventricular mask image. In order to compare the differences of patients (AD and MCI) and normal controls, we subtracted the normal probability map from the patient probability map to obtain the discriminate map. At last, a matching coefficient (MC) between a testing input and the discriminate map is calculated by

(4) MCNormal  or  patienti=∑∀x,y,zD(x,y,z)  ×TNormal  or  patienti(x,y,z),

where *D*(*x*, *y*, *z*) is the discriminate map and *T* denotes the testing ventricular mask image.

#### 2.3.2. D Shape Features

The 2D shape features are extracted from the segmented ventricles on a slice-by-slice basis. In 2D viewpoint, there are many 2D ventricle slices for each case. In order to effectively compare the differences in each case, we selected the slices with maximum areas from 3D ventricle data sets as the datum plane. These 2D shape features used herein are referred to the work of Yang et al. [29] and listed as follows: (1)  *Area*, (2)  *Perimeter*, (3)  *Compactness*, (4)  *Elongation*, (5)  *Rectangularity*, (6)  *Distances*, (7)  *Minimum thickness*, *and* (8)  *Mean signature value*.

### 2.4. Learning Methods for Classification

Machine learning algorithms can be organized into a taxonomy based on the desired outcome of the algorithm or the type of input available during training the machine. They are often divided into supervised, nonsupervised, and reinforcement learning (RL). Supervised learning requires the explicit provision of input-output (I/O) pairs and the task is one of constructing a mapping from one to the other. Non-supervised learning has no concept of target data and performs processing only on the input data. In contrast, RL uses a scalar reward signal to evaluate I/O pairs and hence discover, through trial and error, the optimal outputs for each input. In this sense, RL can be thought of as intermediary to supervised and non-supervised learning since some form of supervision is present, albeit in the weaker guise of the reward signal. As such, the trained algorithm may be treated as a “black box” encapsulating knowledge gleaned from the training data whose inputs are useful for producing the expected outcome. For this reason, machine learning and computer-aided diagnostics (CADs) have been of growing interest in the field of medical applications. To evaluate whether the performance of supervised and non-supervised methods is good or not, we used three classifiers to produce the outcome. 

In many researches of pattern recognition, dataset is often divided into two subsets of training and testing. The former is used to create the model, and the latter is used to assess the accuracy of the model to predict the unknown sample. This method can be called Train-and-Test method. Cross-validation is the experimental method to effectively estimate the generalization error. In this study, leave-one-out cross-validation (LOOCV) is adopted in three classifiers to estimate dependable generalization error. LOOCV involves using a single observation from the original sample as the validation data, and the remaining observations as the training data. In this section, the classifiers we adopted are illustrated in the following subsections particularly.

#### 2.4.1. Self-Organizing Map Architecture

A self-organizing map (SOM) is a type of artificial neural network for the visualization of high-dimensional data. In general, SOMs are divided into two parts: training and mapping. Training builds the map using input examples, called a Kohonen map [30]. An SOM consists of components called nodes or neurons. Each node has a set of neighbors. When this node wins a competition, not only its weight is adjusted, but those of the neighbors are also changed. They are not changed as much though. The further the neighbor is from the winner, the smaller its weight change. Furthermore, as training goes on, the neighborhood gradually shrinks. At the end of training, the neighborhoods have shrunk to zero size.

When a training example is fed to the network, its Euclidean distance to all weight vectors is computed by using (5). Here *n* denotes the dimension of data, and *t* is the index of the data item in a given sequence,

(5) $$\begin{array}{c} x\left(t\right)=\left\{\zeta_{1}\left(t\right),\zeta_{2}\left(t\right),\ldots ,\zeta_{n}\left(t\right)\right\}. \end{array}$$

The neuron with weight vector most similar to the input is called the best matching unit (BMU). The weights of the BMU and neurons close to it in the SOM lattice are adjusted towards the input vector. The magnitude of the change decreases with time and with distance from the BMU. The update formula for a neuron with weight vector is

(6) $$\begin{array}{c} m_{i}\left(t+1\right)=m_{i}\left(t\right)+\alpha \left(t\right)h_{ci}\left(t\right)\left[x\left(t\right)-m_{i}\left(t\right)\right], \end{array}$$

where *α*(*t*) is a monotonically decreasing learning coefficient and *x*(*t*) is the input vector. The neighborhood function *h*_*ci*_(*t*) depends on the lattice distance between the BMU and neuron. The neighborhood function *h*_*ci*_(*t*) is

(7) $$\begin{array}{c} h_{ci}\left(t\right)=\frac{e^{-{||r_{i}-r_{c}||}^{2}}}{2\sigma^{2}\left(t\right)}. \end{array}$$

**Figure 4:**
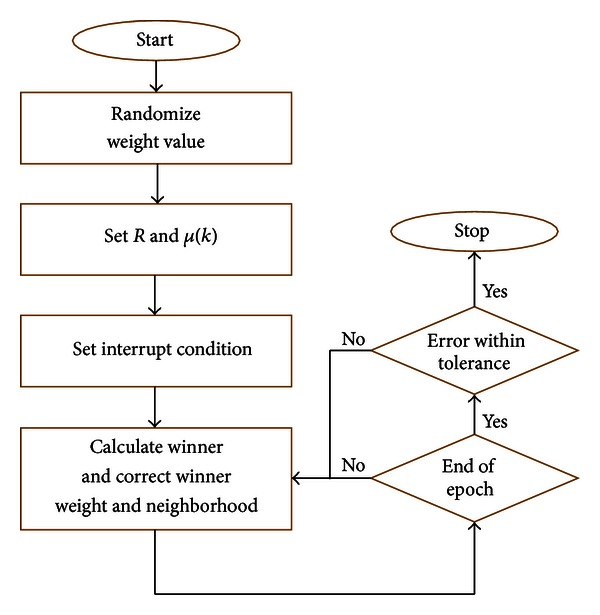
Basic procedure of SOM classifier.

Figure 4 illustrates the procedure of SOM classifier. In this study, we use a two-stage method for learning [31]. First, we adopt less iterative time, higher learning rate, and large neighborhood distance for learning and make it convergence speedily. After repeating many times, we can acquire network parameters which have the best convergence. Next, combining higher iterative time, less learning rate, and small neighborhood distance with network parameters obtained in first stage to conduct second learning and adjust network parameters slowly. At last, we obtain these parameters: iterative time is set as 1000 epochs, ordering phase learning rate = 0.9, tuning phase learning rate = 0.5, and tuning phase neighborhood distance = 0.5. In order to verify the stability of SOM to generalize the correct tendency, the classifier was trained 10 times to get reliable results. Thirty cases are chosen (AD = 7, Normal = 7, MCI = 8) to be the training set randomly. Scaling of variables is of special importance in our model since the SOM algorithm uses Euclidean metric to measure distances between vectors. In order to solve this problem, we achieved this by linearly scaling all variables so that their variances were equal to one.

#### 2.4.2. Support Vector Machine

SVM is a type of artificial neural networks that is, trained by using supervised learning, have shown their advantage on reducing training-and-testing errors, resulting in obtaining higher recognition accuracy [32]. However, some feature data are linearly nonseparable. In some situations, features are not perfectly separable, especially at the border between categories. To allow some flexibility in separating the categories, SVMs utilize a cost parameter, denoted as *C*, to control the trade-off between allowing training errors and forcing rigid margins. The cost function with *C* is defined as (8), where *ζ*_*i*_ is a slack variable,

(8) $$\begin{array}{c} \text{Cost}=C\sum_{i=1}^{N}\left(\zeta_{i}\right). \end{array}$$

Mapping the patterns in a high dimension feature space is generated through combining features to form a kernel matrix. The kernel matrix is usually constructed by using a kernel function which takes two patterns as arguments and outputs a value. In this study, a radial basis function (RBF) kernel, as shown in (9), is employed. We use one-against-rest assembles classifiers that distinguish one from all the other classes. This strategy consists of constructing one SVM per class, which is trained to distinguish the samples of one class from the samples of all remaining classes. Usually, classification of an unknown pattern is done according to the maximum output among all SVMs,

(9) $$\begin{array}{c} k\left(x_{i},y_{j}\right)=e^{-\gamma ||x_{i}-y_{j}||{\text{Fit}}_{p}},\;i=j=1,2,\ldots ,n, \end{array}$$

where *x*_*i*_ denotes the input vector, *y*_*j*_ denotes the *j*th prototype vector, and Fit_*p*_ = correctly − classified/total number of testing data. Finally, the optimal solution can be solved by using Lagrange method,

(10) Lp≡12||w||2+C∑i=1mζi−∑i=1mαi{yi(w·xi+b)−1+ζi},LD≡∑i=1mαi−12∑i=1mαiαjyiyjk(xi,yj),

where ||*w*|| is the Euclidean norm of *w*, *α*_*i*_ that stands for the Lagrange multipliers, *L*_*P*_ is the Lagrange function, and *L*_*D*_ is the dual solution of *L*_*P*_. *C* and *γ* are used to control the trade-off between training errors and generalization ability in SVM with RBF kernel. Therefore, a PSO was utilized to find the optimal combination of *C* and *γ*. 

#### 2.4.3. Hybrid PSO-SVM

Particle swarm optimization (PSO) algorithm [33, 34] uses particles moving in an *m*-dimensional space to search solutions of an optimization problem with *m* variables. In our approach, PSO is initialized and searches for the optimal particle iteratively. Each particle represents a candidate solution. SVM classifier is built for each candidate solution to evaluate its performance. Velocity and position of particles can be updated by

(11) vijt+1=w·vijt+c1 rand1(pbestijt−xijt)+c2 rand2(gbestijt−xijt)xijt+1=xijt+vijt+1,

where *t* is evolutionary generation, *v*_*ij*_ is the velocity of particle *i* on dimension *j*, and *x*_*ij*_ stands for the position of particle *i* on dimension *j*. Inertia weight *w* is used to balance the global exploration and local exploitation, rand_1_ and rand_2_ are random functions, and *c*_1_ and *c*_2_ are personal and social learning factors. As we know, if the number of particles, denoted as *P*, is too large, it might cause the optimization process to be time consuming. On the contrary, if *P* is too small, then it is hard to find the optimal solution due to the limited search area. In the literature [35], it is proven that the optimal solution can be obtained when *P* is between 20 and 40. In this work, the number of the iterations and *P* is set to 200 and 30, respectively. Similarly, the parameters *c*_1_, *c*_2_, and *w* will affect the convergence of optimization process. If they are set too large, it causes the particle velocity to be speedy and thus cannot obtain the optimal solution. On the other hand, it is time consuming to find the optimal solution [36]. Therefore, we set *c*_1_, *c*_2_, and *w* to 2, 2, and 0.8, respectively.

**Figure 5:**
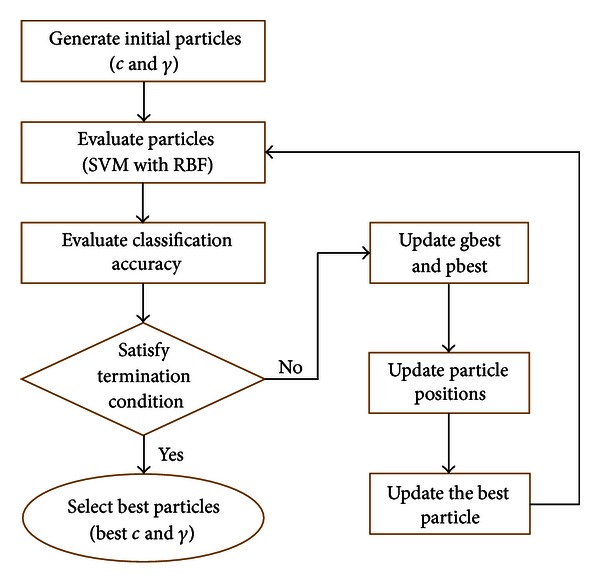
Basic operation of proposed PSO-SVM approach.

More specifically, based on the approach [37], the proposed hybrid PSO-SVM aims at optimizing the accuracy of SVM classifier by randomly generating the parameters (*C* and *γ*) and estimating the best values for regularization of kernel parameters for SVM model. Basic operation of hybrid PSO-SVM proposed in this paper is given in Figure 5. 

This process continues until the performance of SVM converges. The termination criteria are that the iteration number reaches the maximum number of iterations (100%) or the value of global optimal fitness does not improve after 200 consecutive iterations. In this study, 22 cases were chosen (AD = 7, Normal = 7, MCI = 8) to be the training set.

## 3. Experimental Results and Discussion

### 3.1. Materials

According to the research [4], most patients with Alzheimer's disease are aged 65 years or older. Therefore, most of the subjects in the whole data we choose are over 65 years old. The image data used in this study were provided by Chang Gung Memorial Hospital, Lin-Kou, Taiwan. The degree of clinical severity for each participant was evaluated by experienced clinicians whom conducted independent semistructured interviews which included a set of questions regarding the functional status of the participant, along with a standardized neurologic, psychiatric, and health examinations. This interview generates an overall Clinical Dementia Rating (CDR) and Mini Mental State Examination (MMSE) score. The whole dataset consists of three groups comprising normal control, MCI, and AD. Demographic information is provided in Table 1.

The whole-brain MRI scans were obtained by a 3T MR scanner (Trio A TIM system, Siemens, Erlangen, Germany). T1-weighted images were acquired by magnetization-prepared 180 degrees radio-frequency pulses and rapid gradient-echo (T1-MPRAGE) series. The following imaging parameters were used: repetition time (TR) = 2000 ms, echo time (TE) = 4.16 ms, and flip angle = 9 degrees. The results were represented as a 224 × 256 matrix, and slice thickness = 1 mm in 160 slices.

### 3.2. Statistical Analysis and Classification

Through image processing techniques, we obtained individual volume and shape features. In order to confirm whether there is a significant effect of the classification for these features, we use statistical MW test to compare differences between three groups on various features (continuous variables).

The MW test, also called a Mann-Whitney *U* or Mann-Whitney Wilcoxon test, is a nonparametric rank-based test for identifying the difference between populations with respect to their medians or means. The test does not require sample data to be normal (sample > 30), and it is relatively insensitive to the nonhomogeneity of the variance of sample data. The null hypothesis is that the two populations from which samples have been drawn have equal medians or means. The alternatives are that the populations do not have equal medians. The two samples are combined, and all sample observations are ranked from smallest to largest. It was performed on each feature to evaluate its discriminative power, as shown in (12). *U*_obt_ is the smaller value taken from the sum of *U*_1_ and *U*_2_, where *n*_1_ and *n*_2_ are the sizes of the first and second samples, respectively,

(12) $$\begin{array}{c} Z_{U}=\frac{U_{\text{obt}}-\left(n_{1}n_{2}/2\right)}{\sqrt{n_{1}n_{2}\left(n_{1}+n_{2}+1\right)/12}}. \end{array}$$

The *P* values obtained from the tests can provide the probability that a variation would assume a value greater than or equal to the observed value strictly by chance. It is known that the *P* value which is less than the predetermined significance level (0.05) would result in the rejection of the null hypothesis at the 5% (significance) level. All statistical results of volume and shape features we adopted (<0.05) are shown in Table 2, inclusive of three volume features and seventeen shape features.

### 3.3. Results

Although the features we adopted have statistical significance (<0.05) between three groups, some of the features may be redundant or have high correlation. Therefore, principal component analysis (PCA) [38] is used to reduce the dimensionality of a data set consisting of a large number of interrelated variables, while retaining as much as possible of the variation present in the data set. On the other hand, it can also improve the computation time required for classification. This is achieved by transforming to a new set of variables, the principal components (PCs), which are uncorrelated and are ordered so that the first few retain most of the variation present in all of the original variables. In order to effectively represent all the data, we used the PCs that captured 95% total variation in data set. To train a volume-feature-based classification, the first two principal components were adopted. To train a shape-feature-based classification, only the first eight principal components were adopted. When we integrated volume and shape features into classification, the first six principal components were used to stand for all of the features.  Table 3 gives the variances and the coefficients of the PCs, when the analysis is done on the correlation matrix. The symbol * indicates that this PCA coefficient is used as a feature for classification. SOM, SVM, and PSO-SVM were used to train a classifier, and the results were presented in Tables 4, 5, 6, 7, 8, and 9.

It showed the results of accuracy (proportion of all subjects correctly classified), sensitivity (proportion of individuals with a true positive result), and specificity (proportion of individuals with a true negative result) when using different features. The derivations of accuracy, sensitivity, and specificity were expressed in (13), where TP = true positive, TN = true negative, and FP = false positive. Obviously, incorporating shape features, volume features, and PCA provided excellent classification ability than using only one of them,

(13)   Accuracy  (ACC)=(TP  +  TN)(P  +N)Sensitivity  or  true  positive  rate  (TPR)  =TPP=TP(TP  +  FN)Specificity  or  True  Negative  Rate  (TNR)  =TNN=TN(FP  +  TN).

### 3.4. Discussion

In this study, we investigated the feasibility of using anatomical MR images to extract different types of features as a predictive marker for AD and MCI. We employed multiple features and different classifiers to identify the patients with AD and MCI from normal participants. From the results, volumetric analysis, inclusive of gray/white matter, cerebrospinal fluid, and local shape analysis on ventricle, provides significant atrophy information. Especially, the properties of gray matter volume, ventricular area, elongation, mean signature value, and distances show the statistical significance (<0.01). This implies that using the volume and shape features have the potential ability to identify normal control, AD, and MCI.

By combining both the volumetric features and shape features, the classification accuracy of SOM reached up to 76.47% and 66.67% in patients with AD and MCI, respectively. Moreover, with the help of PCA algorithm, the classification result was improved up to 88.24% and 72.22% in patients with AD and MCI, respectively. The classification accuracy of SVM reached up to 76.47% and 77.78% in patients with AD and MCI, respectively. Moreover, with the help of PCA algorithm, the classification result was improved up to 82.35% and 83.33% in patients with AD and MCI, respectively. With the hybrid classification framework based on PSO, the result achieved up to 82.35% and 77.78% in AD and MCI. Moreover, with the help of PCA algorithm, the classification result was improved up to 94.12% and 88.89% in patients with AD and MCI, respectively. According to the results, combining PSO-SVM with statistical analysis and principal component analysis (PCA) would improve the accuracy of classification. 

 It was also noted that the classification ability was significant for AD and normal control than the patients with MCI. MCI is a transitional stage between normal cognitive aging and dementia. Therefore, the characteristics of patients with MCI were similar to AD subjects. On the other hand, the characteristic of patients with MCI was also possibly similar to normal participants. Combination with other features was essential to improve the accuracy of classification ability for patients with MCI in an early stage. 

## 4. Conclusion

In this paper, we compared different methods for the classification of patients with AD and MCI based on anatomical T1-weighted MRI. To evaluate and compare the performances of each method, two classification experiments were performed: CN versus AD and CN versus MCI. It is observed that the volume features and shape features can be integrated to increase classification accuracy with the low computational complexity. Classification results also verify our hypothesis that the combination of multimodal features, including volume and shape features, outperforms a single modality of features, possibly because different features are mutually complementary. Furthermore, it is proven that statistical analysis and PCA can achieve accuracies significantly better than all the features that are adopted. In the performance of classifiers used here, it is shown that PSO-SVM can achieve the best accuracy, sensitivity, and specificity, no matter for CN versus AD and CN versus MCI.

For the moment, the classified results are greater for patients with AD and normal participants than for patients with MCI. It can provide clinically useful information at the large-scale population-based screening studies. The results would be welcomed for prognosticating disease progression and providing an objective evaluation of cognitive rehabilitation treatments for dementing illness.

## Figures and Tables

**Table 1:** Demographic data and cognitive scores.

Group	Normal control	MCI	AD
Individuals (male/female)	17 (10/7)	18 (9/9)	17 (9/8)
Mean age (yrs)	71.43 ± 4.43	72.50 ± 4.00	72.70 ± 3.93
Education time (yrs)	10.17 ± 5.21	8.22 ± 5.25	5.24 ± 5.36
MMSE scores	28.18 ± 1.70	25.06 ± 4.11	13.29 ± 6.69

**Table 2:** Statistical analysis of features.

Features	Mean volume ± SD				
	Normal	MCI	AD	*P* value (NC versus MCI)	*P* value (NC versus AD)
Volume					
*V*_GM_	862.4 ± 42.7	824.6 ± 57.8	789.7 ± 84.3	*0.016 *	*0.007 *
*V*_WM_	637.6 ± 45.8	601.8 ± 21.2	558.1 ± 63.4	*0.021 *	*0.019 *
*V*_CSF_	863.1 ± 112.9	909.7 ± 128.5	971.8 ± 132.5	*0.038 *	*0.017 *
Shape					
Area	1792.4 ± 278.5	1903.5 ± 426.6	2361.1 ± 802.3	*0.029 *	*0.024 *
Area (PR)	673.5 ± 121.5	874.9 ± 132.5	911.4 ± 183.2	*0.024 *	*0.018 *
Area (PL)	647.1 ± 137.2	872.5 ± 142.5	910.9 ± 183.5	*0.031 *	*0.011 *
Area (FR)	151.9 ± 117.6	231.5 ± 162.4	262.4 ± 167.8	*0.020 *	*0.009 *
Area (FL)	162.7 ± 91.0	258.2 ± 144.3	278.5 ± 189.2	*0.022 *	*0.010 *
Perimeter	226.7 ± 23.1	276.9 ± 20.2	289.8 ± 27.6	*0.029 *	*0.019 *
Circularity	45.6 ± 4.9	39.8 ± 3.6	38.2 ± 2.7	*0.039 *	*0.021 *
Elongation	1.1 ± 0.4	1.4 ± 0.6	1.3 ± 0.2	*0.016 *	*0.009 *
Rectangularity	0.5 ± 0.2	0.6 ± 0.4	0.6 ± 0.1	*0.028 *	*0.016 *
*d*(A, G)	37.3 ± 2.1	38.4 ± 3.7	40.6 ± 4.2	*0.031 *	*0.037 *
*d*(B, G)	36.1 ± 1.8	39.2 ± 3.1	43.1 ± 6.1	*0.034 *	*0.028 *
*d*(C, G)	38.6 ± 4.3	41.4 ± 2.9	42.9 ± 4.6	*0.042 *	*0.030 *
*d*(D, G)	34.7 ± 2.9	39.7 ± 1.4	42.8 ± 4.1	*0.022 *	*0.028 *
*d*(A, C)	72.8 ± 4.3	81.7 ± 8.4	83.8 ± 8.4	*0.009 *	*0.011 *
*d*(B, D)	72.5 ± 4.9	78.2 ± 3.1	81.6 ± 8.2	*0.011 *	*0.007 *
Min thickness	27.4 ± 3.8	29.0 ± 2.6	30.1 ± 3.4	*0.020 *	*0.009 *
Mean Sig.	25.6 ± 3.1	27.9 ± 2.7	29.8 ± 3.1	*0.032 *	*0.013 *

**Table 3:** PCs and their proportion of total variation.

Features	No. of principal component							
Proportion (%)	C1	C2	C3	C4	C5	C6	C7	C8
Volume features (2)	64.16*	31.57*	4.27					
Shape features (17)	48.79*	23.39*	9.43*	6.45*	3.28*	2.13*	1.01*	0.73*
Volume + shape (20)	49.31*	19.98*	13.62*	6.93*	4.47*	2.35*	0.99	0.72

**Table 4:** Classification results (SOM).

Proportion	Volume features	Volume features + PCA	Shape features	Shape features + PCA	Volume + shape features	Volume + shape features + PCA
AD (versus NC)						

Accuracy	76.47%	82.35%	64.71%	70.59%	76.47%	88.24%
Sensitivity	81.25%	87.50%	68.75%	70.59%	76.47%	88.24%
Specificity	77.78%	83.33%	66.67%	70.59%	76.47%	88.24%

MCI (versus NC)						

Accuracy	61.11%	66.67%	50.00%	50.00%	66.67%	72.22%
Sensitivity	78.57%	85.71%	64.29%	64.29%	75.00%	86.67%
Specificity	66.67%	71.43%	57.14%	57.14%	68.42%	75.00%

**Table 5:** Confused matrix with SOM (volume + shape/volume + shape + PCA).

	NC	MCI	AD
NC	**13/15**	2/2	1/0
MCI	3/2	**12/13**	3/5
AD	1/0	4/3	**13/15**

**Table 6:** Classification results (SVM).

Proportion	Volume features	Volume features + PCA	Shape features	Shape features + PCA	Volume + shape features	Volume + shape features + PCA
AD (versus NC)						

Accuracy	70.59%	70.59%	58.82%	64.71%	76.47%	82.35%
Sensitivity	70.59%	66.67%	66.67%	78.57%	76.47%	87.50%
Specificity	70.59%	68.75%	63.16%	70.00%	76.47%	83.33%

MCI (versus NC)						

Accuracy	55.56%	61.11%	44.44%	50.00%	77.78%	83.33%
Sensitivity	66.67%	64.71%	61.54%	75.00%	77.78%	88.24%
Specificity	60.00%	61.11%	54.55%	60.87%	76.47%	83.33%

**Table 7:** Confused matrix with SVM (volume + shape/volume + shape + PCA).

	NC	MCI	AD
NC	**13/15**	0/1	1/0
MCI	3/2	**14/15**	3/3
AD	1/0	4/2	**13/14**

**Table 8:** Classification results (PSO-SVM).

Proportion	Volume features	Volume features + PCA	Shape features	Shape features + PCA	Volume + shape features	Volume + shape features + PCA
AD (versus NC)						

Accuracy	76.47%	76.47%	70.59%	76.47%	82.35%	94.12%
Sensitivity	76.47%	76.47%	70.59%	76.47%	87.50%	94.12%
Specificity	76.47%	76.47%	70.59%	76.47%	83.33%	94.12%

MCI (versus NC)						

Accuracy	66.67%	66.67%	55.56%	50.00%	77.78%	88.89%
Sensitivity	75.00%	75.00%	66.67%	69.23%	87.50%	94.12%
Specificity	68.42%	68.42%	60.00%	59.09%	78.95%	88.88%

**Table 9:** Confused matrix with PSO-SVM (volume + shape/volume + shape + PCA).

	NC	MCI	AD
NC	**15/16**	1/1	0/0
MCI	2/1	**14/16**	3/1
AD	0/0	3/1	**14/16**

## References

[1] Gauthier S, Reisberg B, Zaudig M (2006). Mild cognitive impairment. *The Lancet*.

[2] Geslani DM, Tierney MC, Herrmann N, Szalai JP (2005). Mild cognitive impairment: an operational definition and its conversion rate to Alzheimer’s disease. *Dementia and Geriatric Cognitive Disorders*.

[3] Petersen RC (2004). Mild cognitive impairment as a diagnostic entity. *Journal of Internal Medicine*.

[4] http://www.alz.org/alzheimers_disease_facts_figures.asp?type=homepage.

[5] Folstein MF, Folstein SE, McHugh PR (1975). ‘Mini mental state’. A practical method for grading the cognitive state of patients for the clinician. *Journal of Psychiatric Research*.

[6] Hughes CP, Berg L, Danziger WL (1982). A new clinical scale for the staging of dementia. *British Journal of Psychiatry*.

[7] Teng EL, Hasegawa K, Homma A (1994). The cognitive abilities screening instrument (CASI): a practical test for cross-cultural epidemiological studies of dementia. *International Psychogeriatrics*.

[8] Tombaugh TN (2004). Trail Making Test A and B: normative data stratified by age and education. *Archives of Clinical Neuropsychology*.

[9] Padilla P, Górriz JM, Ramírez J (2010). Analysis of SPECT brain images for the diagnosis of Alzheimer’s disease based on NMF for feature extraction. *Neuroscience Letters*.

[10] Chaves R, Ramirez J, Gorriz JM, Puntonet CG (2012). Alzheimers’s Disease Neuroimaging Initiative, Association rule-based feature selection method for Alzheimer’s disease diagnosis. *Expert Systems With Applications*.

[11] Ramírez J, Górriz JM, Segovia F (2010). Computer aided diagnosis system for the Alzheimer’s disease based on partial least squares and random forest SPECT image classification. *Neuroscience Letters*.

[12] Gallix A, Gorriz JM, Ramirez J, Illan IA, Lang EW (2012). On the empirical mode decomposition applied to the analysis of brain SPECT images. *Expert Systems With Applications*.

[13] Salas-Gonzalez D, Gorriz JM, Ramirez J (2012). Two approaches to selecting set of voxels for the diagnosis of Alzheimer's disease using brain SPECT images. *Digital Signal Processing*.

[14] Iglesias JE, Jiang J, Liu CY, Tu Z Alzheimers's Disease Neuroimaging Initiative, Classification of Alzheimer's disease using a self-smoothing operator.

[15] Vemuri P, Gunter JL, Senjem ML (2008). Alzheimer’s disease diagnosis in individual subjects using structural MR images: validation studies. *NeuroImage*.

[16] Zhang D, Wang Y, Zhou L, Yuan H, Shen D (2011). Multimodal classification of Alzheimer’s disease and mild cognitive impairment. *NeuroImage*.

[17] Vemuri P, Wiste HJ, Weigand SD (2009). MRI and CSF biomarkers in normal, MCI, and AD subjects: predicting future clinical change. *Neurology*.

[18] Juottonen K, Laakso MP, Partanen K, Soininen H (1999). Comparative MR analysis of the entorhinal cortex and hippocampus in diagnosing Alzheimer disease. *American Journal of Neuroradiology*.

[19] Colliot O, Chételat G, Chupin M (2008). Discrimination between Alzheimer disease, mild cognitive impairment, and normal aging by using automated segmentation of the hippocampus. *Radiology*.

[20] Morra JH, Tu Z, Apostolova LG (2009). Automated mapping of hippocampal atrophy in 1-year repeat MRI data from 490 subjects with Alzheimer’s disease, mild cognitive impairment, and elderly controls. *NeuroImage*.

[21] Kantarci K (2005). Magnetic resonance markers for early diagnosis and progression of Alzheimer’s disease. *Expert Review of Neurotherapeutics*.

[22] Talairach J, Tournoux P (1988). *Co-Planar Stereotaxic Atlas of the Human Brain*.

[23] Mazziotta J, Toga A, Evans A (2001). A probabilistic atlas and reference system for the human brain: International Consortium for Brain Mapping (ICBM). *Philosophical Transactions of the Royal Society B*.

[24] Fritzsche KH, von Wangenheim A, Abdala DD, Meinzer HP (2008). A computational method for the estimation of atrophic changes in Alzheimer’s disease and mild cognitive impairment. *Computerized Medical Imaging and Graphics*.

[25] http://www.fil.ion.ucl.ac.uk/spm/.

[26] Jiang CF, Huang CH, Yang ST (2011). Using maximal cross-section detection for the registration of 3D image data of the head. *Journal of Medical and Biological Engineering*.

[27] Wang J, Ekin A, De Haan G Shape analysis of brain ventricles for improved classification of alzheimer’s patients.

[28] Wang J, De Haan G, Unay D, Soldea O, Ekin A Voxel-based discriminant map classification on brain ventricles for Alzheimer’s disease.

[29] Yang ST, Lee JD, Huang CH, Wang JJ, Hsu WC, Wai YY (2010). An image-aided diagnosis system for dementia classification based on multiple features and self-organizing map. *Lecture Notes in Computer Science*.

[30] Kohonen T (1990). The self-organizing map. *Proceedings of the IEEE*.

[31] Wu S, Chow TWS (2007). Self-organizing and self-evolving neurons: a new neural network for optimization. *IEEE Transactions on Neural Networks*.

[32] Cortes C, Vapnik V (1995). Support-vector networks. *Machine Learning*.

[33] Kennedy J, Eberhart R Particle swarm optimization.

[34] Cui Z, Wang L, Tan Y (2010). Particle swarm optimization with active congregation. *ICIC Express Letters*.

[35] Kudo M, Sklansky J (2000). Comparison of algorithms that select features for pattern classifiers. *Pattern Recognition*.

[36] Shi Y, Eberhart R Modified particle swarm optimizer.

[37] Tu CJ, Chuang LY, Chang JY, Yang CH (2007). Feature selection using PSO-SVM. *IAENG International Journal of Computer Science*.

[38] Jolliffe IT (2002). *Principal Component Analysis*.

